# Prevalence of Helminths in Dogs and Owners' Awareness of Zoonotic Diseases in Mampong, Ashanti, Ghana

**DOI:** 10.1155/2016/1715924

**Published:** 2016-01-31

**Authors:** Papa Kofi Amissah-Reynolds, Isaac Monney, Lucy Mawusi Adowah, Samuel Opoku Agyemang

**Affiliations:** ^1^Department of Science Education, University of Education, Winneba, P.O. Box M40, Mampong, Ashanti, Ghana; ^2^Department of Environmental Health and Sanitation Education, University of Education, Winneba, P.O. Box M40, Mampong, Ashanti, Ghana

## Abstract

Dogs are popular pets that live closely with humans. However, this cohabitation allows for the transmission of zoonotic parasites to humans. In Ghana, very little is known about zoonotic parasites in dogs. We examined excrements of 154 dogs for intestinal helminthes using saturated sodium chloride as a floatation medium and further interviewed 100 dog owners regarding knowledge on zoonosis and pet management practices. Thirteen parasite species were identified, with an overall prevalence of 52.6%. Nematodes were more common than cestodes, with* Toxocara canis* being the most prevalent helminth (18.8%). Age (*p* = 0.011; *χ* ^2^ = 9.034) and location (*p* = 0.02; *χ* ^2^ = 12.323) of dogs were significant risk factors of helminthic infections, while mode of housing, function, and gender of dogs were not. Knowledge on zoonosis and pet management practices were poor, including irregular deworming and feeding of animals off the bare ground. Dogs may play an active role in the transmission of zoonotic diseases in the area, given the cohabitation of infected dogs with humans; irregular deworming pattern of dogs; and rampant excretion of helminth-infested dog excreta into the environment.

## 1. Introduction

Dogs live in close association with humans, providing them with companionship and security, among others [1]. However, these companion animals can as well transmit diseases to humans who have close contact with them [2, 3]. Infections could be transmitted to humans through contact with animal hair [4, 5], food and water contaminated with dog excreta or secretions, and/or consumption of dog meat [6]. According to the literature, dogs can host well-known zoonotic parasites, including* Toxocara canis*,* Diphyllobothrium latum*,* Ancylostoma* spp.,* Uncinaria stenocephala* [7], and* Echinococcus granulosus* [8]. The presence of these parasites in dogs causes different clinical symptoms depending on the parasite species and density [9].

There are numerous reports on canine intestinal parasites worldwide. Some studies reported prevalence of between 4 and 40% [7, 10–12]. Others reported higher prevalence of over 60% [9, 13–16]. The varying prevalence reported could be due to differences in status of dog sampled, geographical location, and the diagnostic techniques used [17, 18]. Gastrointestinal parasites are more common in dogs in developing countries [19]. High prevalence and heavy infections are often reported in such countries. This is attributed to the fact that dogs in these regions are rarely treated for parasitic diseases and policies on pet ownership are usually lacking [15] or poorly enforced, thereby providing fertile grounds for zoonotic transmission of parasites.

In Ghana, very little attention has been given to parasites in dogs and only two studies have been conducted in this regard. Studies conducted by Anteson and Corkish [20] and Johnson et al. [21] identified a total of 9 species of intestinal helminths in dogs in Ghana. Anteson and Corkish highlighted the ineffectiveness of antihelminthics, while asserting the possible transmission of zoonotic parasites to children [20]. Johnson et al. [21] identified housing styles, sources of dogs, and purpose of keeping dogs as significant factors associated with infection. Data from these studies were however not population-based as they focused only on owned dogs, but not stray or unowned dogs.

To the best of our knowledge, there has been no survey on intestinal helminths in dogs in Mampong, Ashanti. Current epidemiological data is therefore needed for establishing effective control measures in animal and public health. We report for the first time the prevalence and types of helminths in dogs, deworming practices, and knowledge of pet owners on zoonotic parasites in the area.

## 2. Experimental Section

### 2.1. Study Area and Study Design

Mampong, Ashanti, is the capital of the Mampong Municipal Assembly in the Ashanti region of Ghana. Geographically, it is located on latitude 7°05′42′′N and longitude 1°24′49′′W, approximately 60 km northeast of the regional capital, Kumasi. It has an estimated population of 40,000 people, accounting for approximately half of the population of the entire municipality. The town lies within a wet semiequatorial forest zone and has scenic undulating land forms which range from scarps and hills to low lying tropical areas. Farming activities are predominant in the township owing to the fertile soil.

The study area was divided into three sites according to the planning of settlements. Site 1 is a poorly planned settlement with dispersed housing system and poor environmental conditions. Site 2 and Site 3 have better community setup in terms of housing and environmental conditions compared to Site 1.

Dogs were classified into three age groups as puppies (0–6 months), young dogs (>6 months to 12 months), and adults (>12 months) as described by Bone, 1988 (cited in [9]). They were further categorized into stray, semidomestic, and domestic based on a modified description from the one used by Perera et al. [19]. Domestic dogs were the ones with owners, kept under strict confinement, who do not mingle with stray or semidomestic dogs and may or may not be dewormed and/or vaccinated against rabies and other diseases. The semidomestic dogs were the ones who had owners, who mingle with stray dogs and may or may not be dewormed and/or vaccinated against rabies and other diseases. Stray dogs were the free-ranging ones that did not have owners, fed off the streets, and had no deworming and vaccination against rabies and other diseases.

Random house-to-house screening of dogs of all age groups, sexes, housing styles, and functions was conducted between March and July 2015. Stool samples of one hundred and fifty-four (154) dogs were collected in sterile containers labelled with identification data. After collection, samples were taken to the Laboratory of Veterinary Service, Kumasi, and kept frozen until use. With the informed consent of dog owners, a structured questionnaire was also used to assess the dog management practices and owners' awareness of zoonotic canine parasites.

### 2.2. Laboratory Procedure

Stool samples of dogs were analysed for eggs of parasites using saturated sodium chloride solution as a floatation medium. Samples were observed under the light microscope at 10x objective. The parasites were classified according to their species based on existing keys and descriptions [22]. The results were analysed using SPSS version 17 to determine frequencies and percentages. Test for associations was conducted with the Chi-square (*χ* ^2^) test at 5% significance level.

### 2.3. Assessment of Pet Management Practices and Awareness of Zoonotic Diseases

Questionnaires were administered to 100 dog owners who consented to be interviewed. The questionnaires were divided into two distinct sections to capture information on reasons for keeping dog(s), number of dogs kept, knowledge of zoonosis, and pet management practices including deworming frequency, housing and feeding mode, and veterinary care.

## 3. Results and Discussion

### 3.1. Results

**Figure 1:**
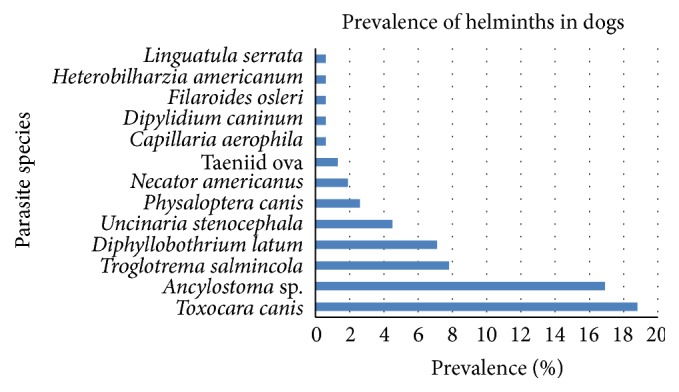
Distribution of helminths found in dog excrement (*n* = 154).

Out of the 154 dog excrement samples examined, approximately 53% were infected with at least one parasite. Overall, 13 parasite species were found in the dog excrement, with the top four parasites being* Toxocara canis* (18.8%),* Ancylostoma* sp. (16.9%),* Troglotrema salmincola* (7.8%), and* Diphyllobothrium latum* (7.1%) (Figure 1). Nematodes were more common than cestodes in the study dogs.

**Figure 2:**
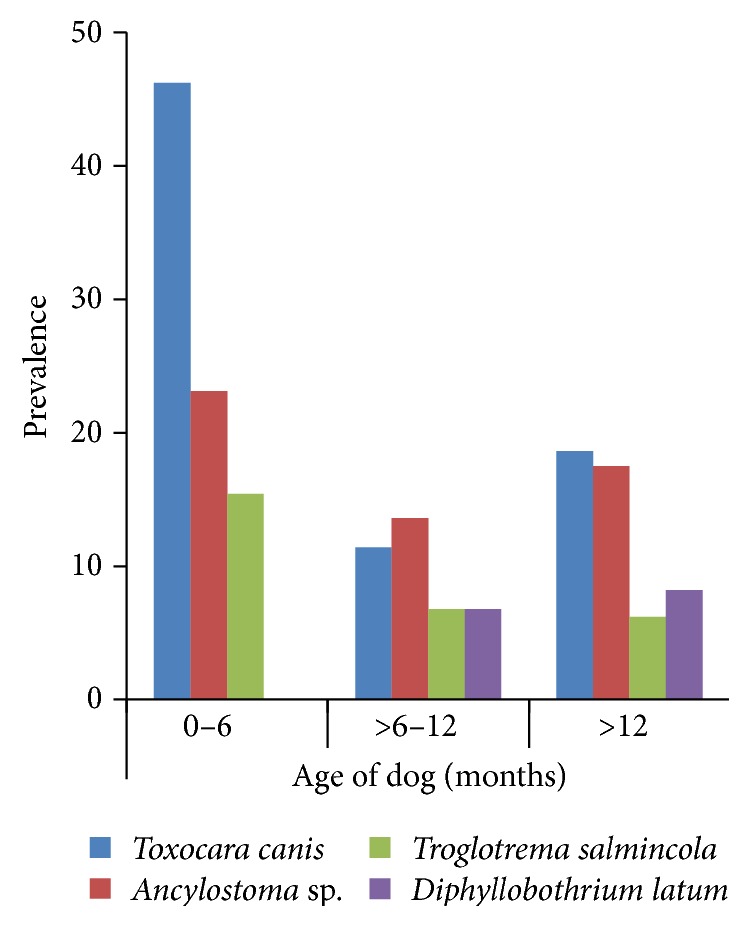
Prevalence pattern of parasites by age.

The prevalence pattern by age of the four most predominant parasites is presented in Figure 2. Only two fish parasites, namely,* Troglotrema salmincola* and* Diphyllobothrium latum*, showed an association with age. While the former showed decreasing prevalence with age of dogs, the latter showed a reverse trend.

The prevalence of helminths in dogs in relation to age and sex is shown in Table 1. Among the three age groups, the highest prevalence (86.7%) was recorded in puppies, followed by adult dogs (52.0%) and young dogs (41.5%). Male dogs recorded slightly higher prevalence (55.1%) than female dogs (48.2%), though the difference was not statistically significant (*p* > 0.05). Eleven (11) species of parasites were recovered from male dogs compared to six (6) from the females.

**Figure 3:**
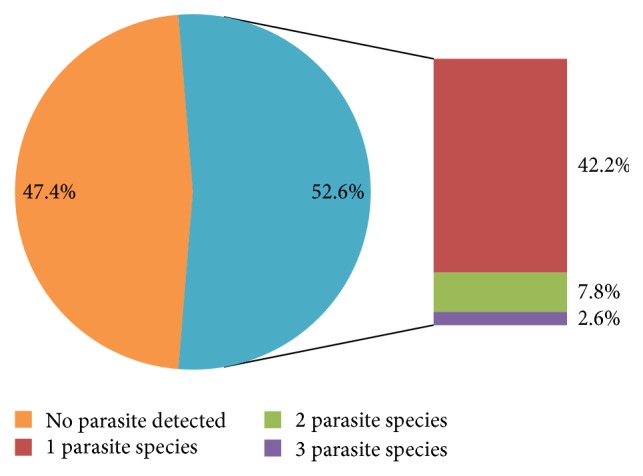
Pattern of parasitic infection among study dogs.

The frequency of single and mixed infections is presented in Figure 3. Single infections were more common (42.2%) than multiple infections (10.4%). The percentage of dogs harbouring mixed infections of two and three parasites was 7.8% and 2.6%, respectively. Interestingly, 13 male dogs (13.2%) harboured multiple parasites compared to 3 female dogs (5.4%). No female dog harboured more than two parasites. Multiple infections were recorded in dogs sampled from Site 1 and Site 2 only.

The prevalence of helminths in relation to the functions of the 145 owned dogs sampled is presented in Table 2. Though dogs who were kept for companionship recorded higher prevalence (69.2%) than dogs used for hunting (66.7%) and security (44.0%), the difference was not statistically significant (*p* > 0.05). Approximately 70% of the owned dogs sampled were kept for security.

Table 3 shows the prevalence of helminths in dogs in relation to their housing styles. Stray dogs recorded the highest prevalence (66.7%), followed by domestic (61.1%) and semidomestic dogs (50.4%), respectively. Nearly 90% of the dogs sampled were allowed to roam about in the community.

A statistically significant association (*p* = 0.02; *χ* ^2^ = 12.323) was found between location and helminthic infections in dogs. The relation between location and prevalence of helminths is shown in Table 4.

None of the 100 dog owners interviewed fed their dogs with standard dog feed and close to three-quarters (73%) fed their dogs off the bare floor (Table 5). Awareness of rabies disease in dogs was comparable (62%) to that of helminth infestation in dogs (60%) among the dog owners. Most dog owners (93%) allowed their dogs to defecate anywhere without any restriction and close to 9 out of 10 dog owners had never taken their dogs to a veterinary clinic although there is one present in the town. Regular deworming of dogs is an uncommon practice among dog owners; close to half of the dog owners had never dewormed their dogs and a significant proportion (76%) had no knowledge of the transmission of zoonotic diseases to humans. About a third of dog owners (32%) kept their dogs in kennels and close to half of them (46%) cleaned the kennels once a month. The median number of dogs kept per owner was 2 (range 1–15).

### 3.2. Discussion

The present study reports for the first time on helminth parasites in dogs in Mampong, Ashanti, Ghana. The two previous studies on dogs in Ghana were done on owned dogs in a different location [20, 21]. All the parasites reported in this study have been previously documented in dogs elsewhere, but with regional variation in prevalence and parasite species.

In the present study, we recorded lower overall prevalence compared to previous data in Africa [9, 13, 15, 16]. The use of single faecal floatation in the present study may have underestimated the prevalence, as a combination of methods has been reported to increase the chances of recovering more parasites [19]. Also, ecological and epidemiological differences, as well as the faecal floatation methods used [11], may account for the variations in distribution and prevalence of parasites. Single infections were more common than multiple infections and this agrees with the findings of Ugbomoiko et al. [15] and Kimura et al. [12]. In contrast, other studies [9, 16, 19] reported higher frequencies of multiple parasites compared to single parasites.

Data from the present study is consistent with previous works in Ghana and other parts of Africa which have reported* Toxocara canis*,* Ancylostoma* sp.,* Dipylidium caninum*, and Taeniidae as some of the helminths parasitizing dogs [9, 13, 15, 16, 21].* Trichuris vulpis*,* Strongyloides* sp., and* Spirocerca lupi* were absent in the present study though these parasites have also been previously reported. The absence of* Spirocerca lupi* eggs in our study may be due to the use of sodium chloride as a floatation medium [9].

Our present results agree with Ugbomoiko et al. [15] and Kimura et al. [12], who reported* Toxocara canis* as the most common helminth in dogs.* Toxocara canis* is a soil-transmitted helminth; thus, habits like feeding off floors and sleeping on bare grounds in the study dogs could account for this observation. Though the prevalence pattern of* Toxocara canis* was not age-dependent, the highest prevalence of this nematode was found in puppies. The older dogs may have developed specific immunity to* Toxocara canis* through frequent exposure at an early age. We also found two fish parasites,* Diphyllobothrium latum* and* Troglotrema salmincola*, in the dogs sampled. Our results also showed that the prevalence pattern for the fish parasites was age-dependent;* Troglotrema salmincola* decreased with age, whereas* Diphyllobothrium latum* showed a reverse trend. The age-dependent prevalence pattern of fish parasites may be due to the role of immune responses in dogs. This role is however unclear and needs to be elucidated. These parasites have only been previously reported in dogs which feed on raw/fresh fish products. Considering that nearly all the dogs sampled fed on raw fish products or viscera, we predict that this prevalence pattern observed is largely due to the feeding habit of the dog rather than the age.

We recorded lower prevalence of* Dipylidium caninum* (0.6%) than has been previously reported elsewhere in Africa. Studies by Anteson and Corkish [20] and Zewdu et al. [9] recorded significantly higher prevalence of* Dipylidium caninum* by postmortem compared to coproscopy. Zewdu et al. [9] further indicated that necropsy provides more detailed information than coproscopy. However, we could not perform necropsy in the present study because the dogs could not be killed for such purposes.

Taeniid tapeworms are morphologically indistinguishable. Therefore, molecular analysis is needed to differentiate species of Taeniidae.* Echinococcus granulosus* is one of the Taeniid species found in dogs, which is also of zoonotic importance. However, the use of coproscopy in the present study did not allow us to detect the species of Taeniidae present.

Age, sex, location, and management practices (including housing styles and deworming practices) are some risk factors that predispose dogs to parasitism. Knowledge of risk factors of infection is vital in the development of effective control programs. Identification of risk factors is however a complex process, particularly in developing countries, given the high numbers of stray dogs with poor or no documented histories. In the present study, age of dog and location were identified as significant risk factors associated with parasitism. Perhaps, the low immunity of puppies compared to the older dogs accounts for the significantly higher prevalence of infection in the former. Though sex was not a significant risk factor, male dogs harboured more parasites species and more multiple infections compared to females. This could be attributed to the greater propensity for male dogs to roam about compared to females.

Ten (10) out of the 13 parasite species encountered were zoonotic, excluding* Physaloptera canis*,* Filaroides osleri*, and* Heterobilharzia americanum*. In addition, majority of the dogs were not regularly dewormed. Deworming of dogs did not appear to be associated with the reasons for keeping the dogs. For some dog owners, deworming was mostly done once a year by veterinary officers during community visits to vaccinate the pets against rabies. In areas where there are strict regulations on pet husbandry practices, dogs are generally given better care. These dogs are more regularly dewormed, mostly kept confined, or always accompanied outside and are not likely to defecate indiscriminately into the environment. These practices have the potential to limit the transfer of zoonotic agents. In comparison with these best practices, veterinary care for dogs in the study area was very poor. Except for yearly vaccination of dogs against rabies by veterinarians, awareness creation on zoonosis and proper pet management practices was virtually nonexistent. With most dogs harbouring zoonotic parasites, having close bonds with dog owners who irregularly deworm their pets, and defecating indiscriminately, public health is threatened as a result of easy transfer of zoonotic parasites into the environment.

Compared to the most recent report on canine helminths in Ghana, we observed a number of similarities. The prevalence rate of 52.6% reported in our study is comparable with the 62.6% prevalence rate reported by Johnson et al. [21] in Accra, Ghana. The highest prevalence of* Toxocara canis* reported in both studies was found in puppies. Again, more than half of the dogs sampled in the two studies were kept for security and with a similar proportion being allowed to roam about in the communities. Most dog owners kept multiple dogs at home (median: 2 and 3, resp.), but pet management practices were poor. Both studies identified zoonotic canine parasites and reported low awareness of dog owners on risk of zoonotic transmission of parasites. Based on these findings, we predict that data from other parts of the country could show a similar trend and this could have serious implications for animal and public health, with dogs playing active roles in zoonotic transmission.

## 4. Conclusions

The study shows that more than half (approximately 53%) of the study dogs (*N* = 154) were infected with helminthic parasites, mostly nematodes. The top four parasites were* Toxocara canis* (18.8%),* Ancylostoma* sp. (16.9%),* Troglotrema salmincola* (7.8%), and* Diphyllobothrium latum* (7.1%). Age of dogs (*p* = 0.011; *χ* ^2^ = 9.034) and location (*p* = 0.02; *χ* ^2^ = 12.323) were significant risk factors of helminth parasitism, while mode of housing, function, and gender of dogs were not. Only close to a quarter (24%) of dog owners had knowledge of transmission of zoonotic diseases to humans and about half (46%) have never dewormed their dogs although most of them (73%) fed their dogs directly off the ground. Dogs in the area are potential agents of zoonotic transmission given direct excretion of helminth-infested excreta into the environment, cohabitation with owners, and poor pet management practices. The indiscriminate excretion of dogs in the environment is a blot on the landscape and poses a potential pollution source for adjoining surface water resources. The lack of awareness on the transmission of zoonotic diseases from dogs to humans and lack of proper veterinary care for the dogs are a serious public health risk. Dog owners need to be educated and veterinary services should be offered on door-to-door basis instead of the conventional centralised mode.

Given the species diversity of parasites in dogs in the region, we recommend the use of broad spectrum antihelminthics in the treatment of helminthiasis. The study needs to be replicated in other parts of the country to give a holistic impression of the spatial variation of helminth infection among dogs across the country.

## Figures and Tables

**Table 1:** Prevalence of helminths in dogs in relation to age and sex (*N* = 154).

Variable	Number examined	Number infected	Infection rate (%)	*p* value
Age (months)				
0–6	15	13	86.7	*p* = 0.011; *χ* ^2^ = 9.034
>6–12	41	17	41.5	
>12	98	51	52.0	
Sex				
Male	98	54	55.1	*p* = 0.410; *χ* ^2^ = 0.678
Female	56	27	48.2	
Total	**154**	**81**	**52.6**	

**Table 2:** Prevalence of helminths in relation to dog function.

Function of dog	Number of dogs examined	Number of dogs infected	Infection rate (%)	*p* value
Companionship	39	27	69.2	*p* = 0.59; *χ* ^2^ = 5.650
Hunting	6	4	66.7	
Security	100	44	44.0	
Total	**145**	**75**	**51.7**	

**Table 3:** Prevalence of helminths in relation to mode of housing.

Mode of housing	Number of dogs examined	Number of dogs infected	Infection rate (%)	*p* value
Domestic	18	11	61.1	*p* = 0.476; *χ* ^2^ = 1.485
Stray	9	6	66.7	
Semidomestic	127	64	50.4	
Total	**154**	**81**	**52.6**	

**Table 4:** Prevalence of helminths based on location of dog.

Location	Number of dogs examined	Number of dogs infected	Infection rate (%)	*p* value
Site 1	48	30	62.5	*p* = 0.02; *χ* ^2^ = 12.323
Site 2	50	32	64.0	
Site 3	56	19	33.9	
Total	**154**	**81**	**52.6**	

**Table 5:** Dog management practices and awareness of zoonotic parasites (*N* = 100).

*Type of feed given to dog*	
Dog feed	0%
Raw meat products and household leftovers	100%
*How do you feed your dog?*	
In a bowl	13%
In a bowl and/or on the floor	14%
On the floor	73%
*Awareness of rabies in dogs *	
Yes	62%
No	38%
*Awareness of helminthic infections in dogs*	
Yes	60%
No	40%
*Where do(es) your dog(s) usually defecate?*	
Within the house	7%
Within and/or outside the house (anywhere)	93%
*Have you ever taken your dog(s) to a veterinary clinic?*	
Yes	12%
No	88%
*Frequency of deworming dog(s)*	
Once every 3 months	16%
Once every 6 months	13%
Once a year	25%
Never	46%
*Awareness of risk of zoonotic transmission of parasites*	
Yes	24%
No	76%
*Do you keep your dogs in kennels?*	
Yes	32%
No	68%
*How often do you clean the kennels?* ^*∗*^	
Daily	22%
Weekly	32%
Monthly	46%

^*∗*^ *n* = 32.
